# Resveratrol differentially regulates NAMPT and SIRT1 in hepatocarcinoma cells and primary human hepatocytes

**DOI:** 10.1186/2049-3002-2-S1-P65

**Published:** 2014-05-28

**Authors:** Susanne Schuster, Melanie Penke, Theresa Gorski, Anja Barnikol-Oettler, Rolf Gebhardt, Wieland Kiess, Antje Garten

**Affiliations:** 1 Center for Pediatric Research Leipzig (CPL), University Hospital for Children and Adolescents, University of Leipzig, Leipzig, Germany; 2 Institute of Biochemistry, Medical Faculty, University of Leipzig, Leipzig, Germany

## Background

Resveratrol, a plant-derived polyphenol, found in red wine and other food products, is reported to possess chemotherapeutic properties in several cancers. We wanted to investigate the molecular mechanisms of resveratrol-induced cell cycle arrest and apoptosis as well as the impact of resveratrol on NAMPT (Nicotinamide phosphoribosyltransferase) and SIRT1 protein function and asked whether there are differences in hepatocarcinoma cells and non-cancerous primary human hepatocytes.

## Materials and methods

HepG2 cells (p53^+/+^), Hep3B cells (p53^-/-^) and primary hepatocytes cultured from non-cancerous human liver tissues were used. Intracellular protein levels of NAMPT, SIRT1, acetylated p53 (K382), phosphorylated p53 (Ser15) and caspase-3 were analyzed by Western Blot whereas extracellular NAMPT (eNAMPT) was measured by eNAMPT ELISA. NAMPT enzymatic activity was quantified using ^14^C-labeled nicotinamide. Intracellular NAD levels were measured by HPLC-Analysis. SIRT1 overexpression studies were performed using pECE-Flag SIRT1 plasmid via electroporation.

## Results

Hepatocarcinoma cells expressed higher levels of SIRT1 and lower amounts of NAMPT compared to primary human hepatocytes. However, basal NAMPT enzymatic activity in HepG2 cells was higher than in primary hepatocytes. In hepatocarcinoma cells, resveratrol led to cell cycle arrest by activation of p53 and increased expression of p21. Induction of apoptosis in hepatocarcinoma cells was p53-independent and mediated by activation of caspase-3. In contrast, human hepatocytes showed no signs of apoptosis after resveratrol exposure. We further revealed that NAMPT activity was affected by resveratrol and was oppositely regulated in hepatocarcinoma cells (-39%) and primary human hepatocytes (+65%). Acetylation of the tumor suppressor p53 (K382), a major target site of SIRT1 deacetylation, was strongly increased in resveratrol-treated HepG2 cells (13.4-fold) suggesting a SIRT1 inhibition. Resveratrol also induced NAMPT release from hepatocarcinoma cells which was associated with increased NAMPT mRNA expression. This effect was absent in primary hepatocytes where resveratrol was shown to function as NAMPT and SIRT1 activator. Moreover, inhibition of NAMPT and SIRT1 activity, by FK866 or by EX527, led to increased acetylation and transcriptional activity of p53 and induced apoptosis. Furthermore, a SIRT1 overexpression significantly decreased p53 hyperacetylation and resveratrol-induced NAMPT release as well as S-phase arrest in HepG2 cells. However, resveratrol-induced apoptosis was not abrogated after SIRT1 overexpression.

## Conclusion

Our study revealed that resveratrol selectively induced p53-independent cell death in hepatocarcinoma cells and differentially regulated NAMPT and SIRT1 in cancer cells and non-cancerous cells. Our data give evidence that in contrast to normal hepatocytes, resveratrol does not act as a NAMPT and SIRT1 activator in hepatocarcinoma cells.

